# Polyphenols and Glycemic Control

**DOI:** 10.3390/nu8010017

**Published:** 2016-01-05

**Authors:** Yoona Kim, Jennifer B. Keogh, Peter M. Clifton

**Affiliations:** School of Pharmacy and Medical Science, University of South Australia, General Post Office Box 2471 Adelaide SA 5000, Australia; yoona.kim@mymail.unisa.edu.au (Y.A.K.); jennifer.keogh@unisa.edu.au (J.B.K.)

**Keywords:** dietary polyphenols, insulin sensitivity, glucose homeostasis, clinical trials

## Abstract

Growing evidence from animal studies supports the anti-diabetic properties of some dietary polyphenols, suggesting that dietary polyphenols could be one dietary therapy for the prevention and management of Type 2 diabetes. This review aims to address the potential mechanisms of action of dietary polyphenols in the regulation of glucose homeostasis and insulin sensitivity based on *in vitro* and *in vivo* studies, and to provide a comprehensive overview of the anti-diabetic effects of commonly consumed dietary polyphenols including polyphenol-rich mixed diets, tea and coffee, chocolate and cocoa, cinnamon, grape, pomegranate, red wine, berries and olive oil, with a focus on human clinical trials. Dietary polyphenols may inhibit α-amylase and α-glucosidase, inhibit glucose absorption in the intestine by sodium-dependent glucose transporter 1 (SGLT1), stimulate insulin secretion and reduce hepatic glucose output. Polyphenols may also enhance insulin-dependent glucose uptake, activate 5′ adenosine monophosphate-activated protein kinase (AMPK), modify the microbiome and have anti-inflammatory effects. However, human epidemiological and intervention studies have shown inconsistent results. Further intervention studies are essential to clarify the conflicting findings and confirm or refute the anti-diabetic effects of dietary polyphenols.

## 1. Introduction

Type 2 diabetes (T2D) is a chronic disease of metabolic dysregulation, most notably abnormal glucose metabolism, accompanied by complications including cardiovascular disease, retinopathy, nephropathy, neuropathy, leg ulcers and gangrene [1]. In 2013, 382 million people were estimated to have diabetes globally and this number is projected to be 592 million by 2035 [2]. Identification of modifiable lifestyle factors including dietary factors that reduce the incidence of T2D is a vital area of research [3,4]. One dietary factor of interest is consumption of polyphenol-rich foods. Dietary polyphenols have been suggested to lower the risk of T2D [5,6,7].

This review aims to summarize relevant epidemiological and clinical studies linking polyphenol-rich foods to the risk of T2D, and to describe the multiple mechanisms of action of polyphenol-rich foods through which these effects are mediated.

## 2. Polyphenols

**Figure 1:**
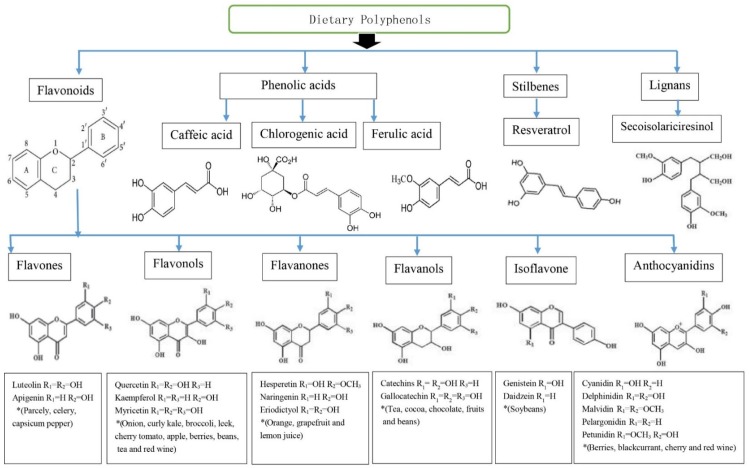
Chemical structures and dietary sources of different groups of polyphenols. Flavonoids are most abundant in four groups of dietary polyphenols and share a basic structure. Resveratrol is one of a subclass of stilbenes. Some phenolic acids are caffeic acid, chlorogenic acid and ferulic acid. Flavones, flavonols, flavanones, flavanols, isoflavone and anthocyanidins are main subclasses of flavonoids. Individual compound of those are characterised in accordance with the arrangement and the number of the hydroxyl groups and their extent of alkylation and/or glycosylation [9]. * Food sources from six main subclasses of flavonoids are briefly described. Typically, quercetin is rich in onion, tea, and apple. Hesperetin is rich in citrus fruits, genistein and daidzein are rich in soybeans. Anthocyanins including cyanidins contribute colour to many red fruits such as strawberry, raspberry and blackcurrant [8,10].

Polyphenols are a large and heterogeneous group of phytochemicals containing phenol rings [6]. Several hundred different polyphenols are found in plant-based foods including vegetables (particularly, broccoli, onion and cabbage), fruits (grapes, pears, apples, cherries and various berries contain up to 200–300 mg polyphenols per 100 g fresh weight), legumes (soybean), cereals, plant-derived beverages and chocolate [5,8,9]. Approximately 100 mg of polyphenols are identified in a cup of coffee or tea or a glass of red wine [8,9]. Polyphenols are divided into flavonoids, phenolic acids, stilbenes, and lignans [9]. Flavonoids include flavones, flavonols flavanols, flavanones, isoflavones, and anthocyanins [7,8,10]. The most common phenolic acids are caffeic acid (present in many fruits and vegetables, most often esterified with quinic acid as in chlorogenic acid, which is a major phenolic compound in coffee) and ferulic acid present in cereals which is esterified to hemicelluloses in the cell wall. The best studied stilbene is resveratrol in grapes, grape products and red wine [10]. The richest source of lignans is linseed present mainly as secoisolariciresinol (up to 3.7 g/kg dry weight) with low quantities of matairesinol, while minor sources are cereals, lentils, fruits (pears, prunes) and vegetables (garlic, asparagus, carrots) [10]. Chemical structures and dietary sources of different groups of polyphenols are shown in Figure 1.

## 3. Epidemiological Studies of Dietary Polyphenols

Epidemiological studies support favourable effects of polyphenol-rich diets in preventing and managing T2D [6].

### 3.1. Flavonoids

A prospective study in 1111 T2D case-control pairs in the Nurses’ Health Study I and II (NHS and NHSII) investigating urinary excretion of eight polyphenol metabolites found that flavanones (naringenin and hesperetin) and flavonols (quercetin and isorhamnetin), as well as the phenolic acid, caffeic acid, were associated with a 39%–48% lower T2D risk during early follow-up (median of ≤4.6 years since urine sample collection), but not during later follow-up which ranged from 4.6 to 11.4 years [11]. Metabolites of flavan-3-ols and ferulic acid were not associated with T2D risk in either period [11]. This is in accordance with the Women’s Health Study of 38,018 US women aged ≥45 years with an average 8.8 years of follow-up which reported that none of total flavonols and flavones, or quercetin, kaempferol, myricetin, apigenin, and luteolin were significantly associated with the risk of T2D [12]. However, apple consumption of ≥1 apple/day showed a 28% lower T2D risk compared with no apple consumption (relative risk—RR 0.72; 95% confidence interval (CI) 0.56–0.92; *p* = 0.006) [12]. A Finnish study of 10,054 men and women with 526 T2D cases showed that intakes of apples (hazard ratio—HR 0.73; 95% CI 0.57–0.92; *p* = 0.003) and berries (HR 0.74; 95% CI 0.58–0.95; *p* = 0.03) were significantly associated with a lower risk of T2D [13]. These findings are consistent with prospective studies of 159,560 women in the NHS and NHS II, and 41,334 men in the Health Professionals Follow-Up Study [14], which also found that higher intakes of anthocyanins were significantly associated with a lower risk of T2D (HR 0.85; 95% CI 0.80–0.91; *p* < 0.001) [14]. Apples/pears (HR 0.77; ≥5 servings/week *vs.* <1 serving/month; 95% CI 0.65–0.83; *p* < 0.001) and blueberries (HR 0.77; ≥2 servings/week *vs.* <1 serving/month; 95% CI 0.68–0.87; *p* < 0.001) were inversely associated with T2D [14], but total flavonoid intake or other flavonoid subclasses were not associated with a lower risk of T2D [14]. An inverse association remained after adjustment for body mass index (BMI) or weight [13,14].

The Iowa Women’s Health Study of mainly white, postmenopausal women did not observe any T2D-protective effect of total flavonoid intake or any of the flavonoid subclasses, including anthocyanins. On the other hand, regular red wine consumption of ≥1 occasion/week was associated with a 16% lower T2D risk than wine consumption <1 occasion/week (HR 0.84; 95% CI 0.71–0.99), with similar findings for white wine, beer, and liquor [15], suggesting it is the alcohol *per se* and not the polyphenols which is associated with protection. Inconsistent findings might be explained due to the use of the older, less complete versions of the United States Department of Agriculture (USDA) database [12,15] and misclassification of intake in the baseline questionnaires [14]. Much of the epidemiological data is based on reported food intake rather than blood or urine measures of polyphenol metabolites and is thus relatively unreliable compared with more objective measures.

### 3.2. Phenolic Acids

Epidemiological studies showed a fairly consistent association between coffee (caffeinated and decaffeinated) [16,17,18,19,20] or green tea [21] consumption and a lower risk of T2D in a dose-response manner in most studies [16,17,18,19,20,21] except [22].

A meta-analysis of 28 prospective studies with 1,109,272 participants and 45,335 T2D cases showed an inverse association of coffee consumption with T2D in a dose-response manner (1–6 cups/day) [16]. Coffee consumption of six cups/day was associated with a 33% lower risk of T2D, compared with no coffee consumption (RR 0.67; 95% CI 0.61–0.74; *p* < 0.001). Both caffeinated and decaffeinated coffee were associated with a lower risk of T2D [16]. A retrospective cohort study of 17,413 Japanese men and women aged 40–65 years, based on completed five-year follow-up questionnaire found RRs of 0.67 (95% CI 0.47–0.94) for green tea intake of ≥6 cups/day and 0.58 (95% CI 0.37–0.90) for coffee intake of ≥3 cups/day, compared with less than one cup/week [21]. The consumption of black or oolong teas was not associated with the risk of T2D [21]. Total caffeine intake was associated with a 33% lower risk of T2D [21]. These inverse associations were more prominent in women and in overweight men [21]. Similarly, another study [17] also observed a stronger inverse association between coffee, decaffeinated coffee and caffeine intake, and T2D incidence in women than in men as well as a stronger inverse association between coffee intake and T2D incidence in non-smokers and subjects with BMI <25 kg/m^2^ [17] compared with smokers and overweight and obese people. Three other meta-analyses showed that tea intakes of ≥3 cups/day (RR 0.84; 95% CI 0.73–0.97) [23] or ≥4 cups/day [24] had a 16% reduction in the risk of developing T2D, compared with the lowest intake (RR 0.84; 95% CI 0.73–0.94) but a third meta-analysis [25] showed no effect.

A prospective cohort of 40,011 participants with a mean follow-up of 10 years and 918 cases of T2D [22] reported potential beneficial effects of both coffee and tea intakes, but there was no dose-response as coffee and/or tea intakes of at least three cups/day reduced the risk of T2D by 42%. Coffee intake of 4.1–6.0 cups/day and tea intake of >5.0 cups/day reduced the risk of T2D by 23% (HR 0.77; 95% CI 0.63–0.95; *p* = 0.033) and 37% (HR 0.63; 95% CI 0.47–0.86; *p* = 0.002), respectively [22]. The relative amount of coffee *vs.* tea was not important [22].

Positive effects of coffee and tea on a lower risk of T2D were independent of weight or BMI [16,18,20,21,22,24].

Observational studies have shown that higher coffee consumption was associated with decreased fasting insulin [26] and 2-h post-load glucose [27], and homeostatic model assessment levels [26,28,29], increased insulin sensitivity [30], but other studies have shown no association of coffee consumption with fasting insulin [27,31] or homeostasis model assessment of insulin resistance (HOMA–IR) [31]. Green tea consumption was positively associated with HOMA–IR (*p* = 0.02) with no dose-response [28]. The Whitehall II study, a British cohort study, showed no association between coffee, tea and the risk of T2D, although there was an association between combined intake of tea and coffee (≥2 cups/day of both beverages) and the risk of T2D (HR 0.68; 95% CI 0.46–0.99; *p* < 0.05) [32].

## 4. Potential Mechanisms of Action of Polyphenols in Diabetes Risk

The hypoglycemic effects of dietary polyphenolic compounds may be related to inhibition of carbohydrate digestion by inhibiting salivary and pancreatic α-amylase and α-glucosidase in the small intestinal brush border, inhibition of glucose absorption, and stimulation of insulin secretion and protection of pancreatic β-cells against glucotoxicity. Polyphenols may suppress glucose release from the liver, and improve glucose uptake in peripheral tissues by modulating intracellular signalling [6]. Polyphenols have antioxidant activity and can inhibit advanced glycation endproduct (AGE) formation [5].

### 4.1. Carbohydrate Digestion and Glucose Absorption in the Intestine

The key enzymes involved in digestion of dietary carbohydrate are α-amylase and α-glucosidase. Maltose, maltotriose and α-dextrins are the three major products of α-amylase digestion. Further digestion occurs in the small intestine by α-glucosidase which is a class of brush-border bound enzymes which hydrolyse the terminal α-1, 4-linked glucose residues [6,33]. Glucose is taken into cells via transporters, predominately sodium-dependent glucose transporter (SGLT1) located in the brush border membrane at the apical side of enterocytes [34].

Inhibition of α-amylase and α-glucosidase activities *in vitro* has been demonstrated with dietary polyphenols from berries (strawberries, raspberries, blueberries and blackcurrants) [35], vegetables (pumpkin, beans, maize and eggplant) [36,37], black rice [38], legumes [39], green and black tea [33], tea polyphenols [40] and red wine [35,41]. Inhibition of glucose transport has been shown with flavonoids and phenolic acids [42,43,44,45,46].

### 4.2. Tissue Uptake of Glucose

Enhanced insulin-mediated glucose uptake *in vitro*, a glucose transporter 4-mediated process, has been shown with dietary polyphenols including epicatechin [47], epigallocatechin-3-*O*-gallate (EGCG) [48], grape seed-derived procyanidins [49,50], bitter melon [51], blueberry [52], canna indica root [53] and black soy bean [54]. Black soybean seed rich in anthocyanins (cyanidin 3-glucoside) and procyanidins (PCs) lowered glucose levels and improved insulin sensitivity by activation of 5′ adenosine monophosphate-activated protein kinase (AMPK) in the skeletal muscle and liver of type 2 diabetic mice. This activation was accompanied by the up-regulation of glucose transporter 4 GLUT4 in skeletal muscle and the down-regulation of gluconeogenesis in the liver in type 2 diabetic mice [54]. EGCG maintained insulin sensitivity in rat L6 muscle cells exposed to dexamethasone and dose-dependently increased glucose uptake and GLUT4 translocation through activation of phosphoinositide 3-kinase (PI3K) signalling and AMPK and PI3K activation [48]. Cinnamon has shown potential to promote insulin signalling, and GLUT4 translocation in Swiss albino mouse embryo fibroblast line 3T3-L1 adipocytes [55], and increase glucose uptake in insulin resistant rats induced by a high fructose diet [56,57].

### 4.3. Gut Microbiota

Only 5%–10% of the total intake of dietary polyphenols are directly absorbed through the stomach and the small intestine [58]. The majority of the ingested polyphenols reach the colon, thereafter undergoing intensive metabolism prior to absorption [58,59]. Some polyphenols are thought to exert a prebiotic effect by stimulating the growth and activity of some bacteria in the digestive tract [59]. After absorption, polyphenols undergo phase I and II biotransformation (sulfation, glucuronidation, methylation and glycine-conjugation) by enterocytes in the liver to increase hydrophilicity favouring urinary secretion [58]. Polyphenol metabolites derived from liver metabolism interact with adipose tissue, pancreas, muscle and liver, and may exert anti-diabetic effects [59]. Absorption of polyphenols can be affected by dosage, size of phenolic compound, prior diet, food matrix, gender and differences in the gut microbial populations [59].An increased level of faecal *Bifidobacteria* has been associated with improved glucose tolerance and diminished inflammatory markers such as the interleukins IL-6, IL-1α and IL-1β, tumor necrosis factor α and monocyte chemoattractant protein-1 [59,60,61]. A few clinical trials [62,63,64,65,66,67] have shown the potential prebiotic effects of dietary polyphenols to increase the population of *Bifidobacteria*. In a randomized, controlled, double-blind, crossover study of 22 health volunteers [62], the daily consumption of 494 mg cocoa flavanols for four weeks significantly increased the growth of *Lactobacillus* spp. (*p* < 0.001) and *Bifidobacterium* spp. (*p* < 0.01) relative to that of a drink containing only 29 mg flavanols and matched for fibre and other potential prebiotics.A significant reduction in C-reactive protein (CRP) (*p* < 0.05) was observed and changes in CRP were associated with changes in *Lactobacilli* counts (*p* < 0.05; *R*^2^ = 0.20) [62]. Similarly, the daily consumption of red wine polyphenols for four weeks modulated the growth of gut microbiota [66] by significantly increasing *Bifidobacterium* and *Enterococcus*, *Prevotella*, *Bacteroides*, *Bacteroides uniformis*, *Eggerthella lenta*, and *Blautia coccoides-Eubacterium* *rectale* groups compared with baseline but there was no control drink for comparison. Improvements in cholesterol and CRP levels were associated with changes in the *Bifidobacteria* amount [66]. Daily consumption of 25 g of wild blueberry powder drink for six weeks increased *Bifidobacterium* spp. compared with the placebo drink (250 mL of water, 7.5 g of fructose, 7 g of glucose, 0.5 g of citric acid, 0.03 g of blueberry flavour and colors) [63]. A proanthocyanidin-rich extract from grape seeds administered to nine healthy adults for two weeks significantly increased *Bifidobacterium* but no control material was provided to volunteers [64].

### 4.4. Pancreatic β-Cell Function

Glucose-stimulated insulin secretion from pancreatic β-cells involves glucose entrance via glucose transporter 2 (GLUT2), increased ATP content through glycolysis and tricarboxylic acid cycle (TCA) activity, and then inactivation of ATP-sensitive K^+^ (KATP) channels on the cell membrane [6,68]. Rat pancreatic β-cells, RIN-m5F treated with EGCG and the buckwheat flavonoid, rutin showed reduced glucotoxicity with activation of insulin receptor substrate 2 (IRS2) and AMPK signalling and elevated adenosine triphosphate (ATP) levels [69]. Activation of AMPK plays a critical role in the protection of β-cell against glucolipotoxicity [70]. The anti-diabetic drugs, metformin and thiazolidinediones (TZDs) are known to activate AMPK [69]. Chronic high glucose exposure directly increased intracellular reactive oxygen species (ROS) generation and disturbed mitochondrial function, uncoupling ATP generation and impairing insulin secretion [69]. Moreover, chronic high glucose exposure suppressed AMPK activity leading to increased lipogenesis [69]. This consequently resulted in impairing normal β-cell functioning [69].

Quercetin, apigenin, and luteolin inhibited cytokine-induced pancreatic β-cell damage through suppression of nuclear factor kappaB (NF-κB) activation in RINmF5 cells [71]. Quercetin showed protective effects by decreasing oxidative stress with preservation of pancreatic β-cell integrity in streptozotocin (STZ)-induced diabetes in rats [72]. Isolated rat islets exposed to (−) epicatechin (0.8 mmol/L) or quercetin (0.01 mmol/L) showed enhanced insulin secretion by approximately 44%–70% in the presence of 20 mmol/L glucose [73].

### 4.5. Liver Glucose Homeostasis

The liver is a key regulator of blood glucose levels in coordination with muscle and adipose tissues [6]. In the postprandial state, the liver stores glucose as glycogen via the glycogenesis pathway involving glucokinase (GK) which is a key enzyme in the regulation of glucose utilization in the liver, and glycogen synthase (GS). Under fasting conditions, the liver produces glucose via two routes—either glycogenolysis or gluconeogenesis involving pyruvate carboxylase, phosphoenolpyruvatecarboxykinase (PEPCK), fructose-1,6-bisphosphatase, and glucose-6-phosphatase [6].

Suppressed hepatic glucose production appears to account for decreased glucose levels in EGCG and green tea feeding and *in vitro* studies [74,75,76]. In the rodent study [76] of EGCC supplementation of either 0.25%–1% for seven weeks, a decrease of glucose levels in EGCC-treated db/db mice was observed in a dose dependent manner, compared with placebo-treated mice [76]. The GK mRNA expression was increased in the livers of mice supplemented with EGCC for seven weeks in a dose dependent manner and a decrease of PEPCK mRNA expression was observed in adipose tissue of EGCC supplemented mice. High-dose EGCC supplementation also increased acyl CoA oxidase-1 (ACO–1) and carnitine palmitoyl transferase-1β (CPT-1β) in both livers and adipose tissues [76]. Moreover, in H4IIE hepatoma cells, EGCC downregulated genes involved in gluconeogenesis and the synthesis of fatty acid, triacylglycerol and cholesterol, whereas genes involved in glycolysis (phosphofructokinase) and glucose transport (glucose transporter 1) were increased [76]. Supplementation of the soy isoflavones genistein and daidzein (0.02% in diet) improved glucose homeostasis in NOD mice with an increase in insulin/glucagon ratio and C-peptide level with preservation of insulin staining β-cell of pancreas. In the liver, glucose-6-phosphatase (G6Pase), PEPCK activities and β-oxidation of fatty acid were suppressed and lipogenesis was increased compared with the control group [77]. In db/db mice, the citrus flavonoids (hesperidin and naringin) were also found to lower glucose levels through up-regulating the hepatic GK via peroxisome proliferator-activated receptor gamma (PPAR γ), and upregulating adipocyte GLUT4. Naringin also suppressed PEPCK and G6Pase. This mechanism of citrus flavonoid is very similar to the mechanism of thiazolidinediones [78]. Similarly, areca nut procyanidins fed 10 mg/kg to multiple low dose streptozocin (MLD–STZ) treated mice for four weeks reduced fasting blood glucose, PEPCK and G6Pase and activated AMPKα [79].

In the fed state, dietary cyanidin 3-glucoside was shown to downregulate G6Pase and PEPCK by enhancing the phosphorylation of Akt and, forkhead box protein O1 (FOXO1—an important transcription factor) and by decreasing the nuclear translocation of FOXO1 in the liver and adipose tissues of high-fat diet-fed and db/db mice [80].

### 4.6. Inflammation

Low-grade inflammation and obesity-induced oxidative stress in the mitochondria may be partly responsible for insulin resistance. Elevated proinflammatory cytokines can alter metabolic homeostatic control systems leading to T2D [81]. Grape-seed procyanidins inhibited the production of proinflammatory molecules such as CRP, IL-6 and TNF-α and enhanced the production of adiponectin in fat fed rats [82]. Dietary cyanidin 3-glucoside (0.2% in diet) improved insulin sensitivity in both high-fat diet fed and obese db/db mice [80], decreased mRNA abundance of inflammatory cytokines including TNF-α, IL-6 and MCP-1 and suppressed macrophage infiltration in adipose tissue [80]. Procyanidins reduced gene expression of NF-κB, cyclooxygenase-2 protein (COX2), CRP, IL-6 and TNF-α in adipocytes [83,84], muscle [84] and liver [85,86] in both rats and mice. In male C57Bl/6j mice, eight-week supplementation of a high-fat diet with an anthocyanin-rich, 4% blueberry powder attenuated gene expression in macrophages in adipose tissue, for TNF-α, IL-6, MCP-1, inducible nitric oxide synthase, and improved whole body insulin sensitivity [87]. Quercetin also showed anti-inflammatory effects in male C57Bl/6j mice [88] and in obese Zucker rats [89]. Supplementation of a high-fat diet with 0.8% quercetin for eight weeks decreased interferon-γ, interleukin-1α, and interleukin-4 in male C57Bl/6j mice [88]. Administration of both 2 and 10 mg/kg of body weight of quercetin for 10 weeks improved insulin sensitivity and blood pressure in obese Zucker rats, but only the dose of 10 mg/kg reduced NOx concentration and visceral adipose tissue (VAT) TNF-α production, increased adiponectin and downregulated fat iNOS expression [89].

**Figure 2:**
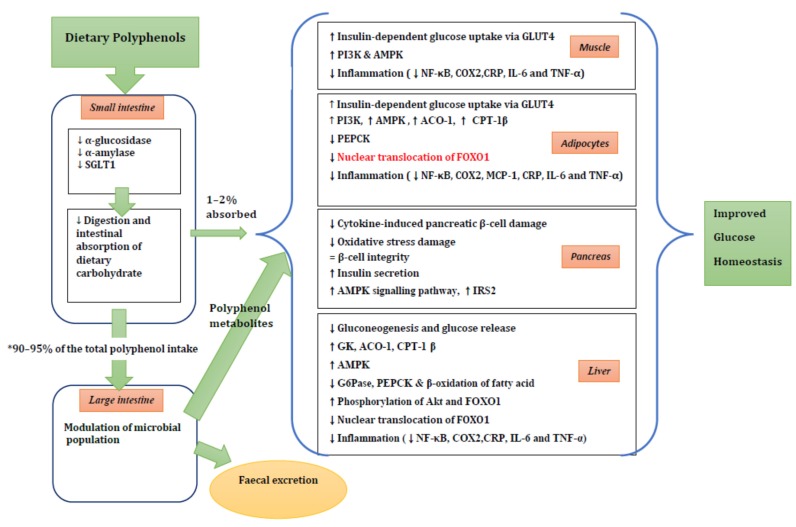
The summary of potential mechanisms linking dietary polyphenol metabolites to improved glucose homeostasis. ↑, increase; ↓, decrease; ↔, maintenance of stability. * 90%–95% of the ingested polyphenols reach the colon. See the text for more details. SGLT1, sodium-dependent glucose transporter; GLUT4, glucose transporter 4; PI3K, phosphoinositide 3-kinase; AMPK, 5′ adenosine monophosphate-activated protein kinase; NF-κB, nuclear factor kappaB; COX2, cyclooxygenase-2 protein; CRP, C-reactive protein; IL-6, interleukin 6; TNFα, tumor necrosis factor α; ACO-1, acyl CoA oxidase-1; CPT-1 β, carnitine palmitoyl transferase-1β; PEPCK, phosphoenolpyruvate carboxykinase; FOXO1, forkhead box protein O1; MCP-1, monocyte chemoattractant protein-1; IRS2, insulin receptor substrate 2; GK, glucokinase; AC0-1, acyl CoA oxidase-1; G6Pase, glucose-6-phosphatase.

A summary of possible mechanisms of action is shown in Figure 2.

## 5. Clinical Studies of Dietary Polyphenols

There are a few clinical studies investigating the effects of specific polyphenols or food products rich in polyphenols in reducing the risk of insulin resistance [6].

### 5.1. Tea and Coffee

In a meta-analysis of green tea or green tea extract of seven randomised controlled trials, the consumption of green tea did not decrease the levels of fasting plasma glucose, fasting serum insulin, 2-h oral glucose tolerance test (OGTT), glucose, glycated hemoglobin (HbA1c) or HOMA–IR index [90]. However the authors stated that limitations of the studies included the small sample sizes, poor quality and low evidence levels of the included studies. In contrast, another meta-analysis of 17 randomised controlled trials including seven high quality studies and 10 low quality studies, demonstrated that green tea had favourable effects significantly reducing the fasting glucose and HbA1c by 0.09 mmol/L (95% CI 0.15–0.03 mmol/L; *p* < 0.01) and 0.30% (95% CI 0.37%–0.22%; *p* < 0.01), respectively. Subgroup analyses from studies with high Jadad scores showed a significant reduction in fasting insulin (−1.16 μIU/mL, 95% CI −1.91 to −0.40 μIU/mL; *p* = 0.03) [91]. This study attributed these findings to measures of glucose control or insulin sensitivity not coming from primary outcomes, limited high quality studies, and concealment of null findings in most selected studies [91].

A third meta-analysis of 22 randomized controlled trials with 1584 subjects showed that green tea catechins (EGCG capsules or powder, whole green tea or green tea extract powder) with or without caffeine reduced fasting glucose (−1.48 mg/dL; 95% CI −2.57 to −0.40 mg/dL), whereas fasting insulin, HbA1c, and HOMA–IR were not affected. In subgroup analyses, the glucose-lowering effect was observed when the duration of follow-up was over a median of 12 weeks. This finding suggested that longer trials were required to support favourable effects of green tea consumption on glycemic control [92].

In summary, given the mixed results from interventions in contrast to epidemiological studies, it is still unclear if polyphenol-rich green tea has anti-diabetic effects.

A randomized cross over study investigating the effect of a single dose of espresso caffeinated coffee, decaffeinated coffee, or water on glucose tolerance and insulin sensitivity (Matsuda index) during an OGTT in 18 habitual coffee drinkers with T2D showed no difference in area under the curve AUC insulin, glucose or insulin sensitivity [31]. However, a three-way, randomized, crossover study of nine healthy subjects [93] showed that chlorogenic acid in coffee acutely modulated gastrointestinal hormone secretion and glucose uptake [93]. The consumption of 25 g glucose in either 400 mL water (control) or 400 mL caffeinated or decaffeinated coffee (equivalent to 2.5 mmol chlorogenic acid/L), did not affect glycemia or insulinemia but attenuated the postprandial increase of glucose-dependent insulinotropic-polypeptide (GIP) (*p* < 0.005) while glucagon-like peptide 1 secretion increased 0–120 min (*p* < 0.01) with a delayed intestinal glucose absorption compared with the control [93]. This finding suggested that chlorogenic acid in coffee decreased the rate of intestinal absorption of glucose or slowed gastric empting [93].

Overall, there is little evidence available to assess the effect of coffee on glucose homeostasis.

### 5.2. Chocolate and Cocoa

Meta-analyses of acute or short-term, chronic (≤18 weeks), randomized controlled trials showed that chocolate or cocoa reduced fasting insulin (−2.65 µU/mL) and insulin after glucose challenge (−17 µU/mL at 30 min; 95% CI −20.7 to −13.4 µU/mL) and improved insulin resistance (HOMA–IR −0.67; 95% CI −0.98 to −0.36). There was no effect on fasting glucose and HbA1c [94]. Dark chocolate (100 g bar containing approximately 500 mg of polyphenols) for 15 days decreased blood pressure and improved insulin sensitivity in healthy individuals as demonstrated by decreasing HOMA–IR (0.94 ± 0.42 *vs.* 1.72 ± 0.62; *p* < 0.001) and increasing quantitative insulin sensitivity check index (QUICKI) (0.398 ± 0.039 *vs.* 0.356 ± 0.023; *p* = 0.001), compared with 90 g white chocolate bar containing no polyphenols [95], and similar results were shown in hypertensive [96] and glucose-intolerant hypertensive individuals [97]. A four-week consumption of dark chocolate containing 500 mg polyphenols reduced blood pressure (BP), fasting glucose and HOMA–IR in lean and overweight females [98] compared with 20 g of placebo dark chocolate containing negligible polyphenol but similar nutritional composition. Consumption of cocoa flavanols (902 mg) for 12 weeks also improved insulin sensitivity in overweight and obese individuals compared with a low-flavanol cocoa drink [99]. In contrast, consumption of flavanol-rich cocoa drink (150 mL twice a day, containing approximately 900 mg flavanols) for two weeks did not improve insulin resistance or blood pressure in people with essential hypertension [100]. Daily consumption of 25 g dark chocolate for eight weeks did not improve fasting glucose, insulin and HbA1c levels in hypertensive diabetic subjects compared with 25 g white chocolate consumption [101]. Similarly, in acute studies [102,103], dark chocolate containing 450 mg of polyphenols did not improve insulin and glucose in hypertensive diabetic subjects given a 75 g oral glucose load 60 min after ingestion of either low (control) or a high polyphenol dark chocolate [102], and cocoa beverage containing 960 mg total polyphenols and 480 mg flavanols showed no effect on glucose and increased insulin levels in obese subjects with T2D given a high-fat breakfast (766 kcal; 50 g fat) compared with a placebo (110 mg total polyphenols; <0.1 mg flavanols) [103].

In summary, given conflicting results in interventions, current data is insufficient to recommend chocolate and cocoa for glycemic control. Therefore, large placebo controlled trials with good quality designs are required to clarify the effects of cocoa polyphenols.

### 5.3. Cinnamon

Cinnamon is known to contain polyphenols such as catechin, procyanidin, cinnamtannin trans-cinnamic acid and flavones (cinnamaldehyde and trans-cinnamaldehyde) [5,104,105]. Eleven studies have shown positive effects of cinnamon on glycemic control. Three studies [106,107,108] included subjects with T2D with baseline HbA1c levels >8%. Seven studies [109,110,111,112,113,114,115] included healthy normal subjects [111,112,113,114], subjects with impaired fasting glucose [109] and subjects with metabolic syndrome [115]. One study showed beneficial effects on improved insulin sensitivity in patients with polycystic ovary syndrome [105]. Two studies [116,117] showed positive effects on fasting blood glucose levels, but did not report baseline HbA1c levels [116] and no significant changes of HbA1c, total cholesterol, low-density lipoprotein (LDL), high-density lipoprotein (HDL) or TG [117]. Five studies [118,119,120,121,122] showed no significant changes in fasting glucose, lipids, HbA1c, or insulin levels in 25 postmenopausal women with T2D taking 1.5 g of cinnamon daily for six weeks [120], in 43 subjects with T2D receiving 1 g of cinnamon daily for three months [118], in 60 subjects with T2D taking 1 g of cinnamon daily for 12 weeks [121], in 72 adolescents with Type 1 diabetes (T1D) taking 1 g of cinnamon daily [122] and in 11 healthy subjects taking 3 g of cinnamon daily for four weeks [119]. A randomized, controlled trial [108] (no placebo used with the concern that the strong taste of cinnamon capsules would preclude any blinding), investigating whether daily cinnamon plus usual care *versus* usual care alone lowers HbA1c in 109 patients with T2D (HbA1c > 7.0), showed that supplementation with 1 g of daily cassia cinnamon did not significantly lower HbA1c over 90 days [108]. A randomized, placebo-controlled, double-blind clinical trial of 58 subjects with T2D found that intake of 2 g daily of cinnamon for 12 weeks significantly reduced HbA1c (−0.36%; *p* < 0.005), systolic blood pressure—SBP (−4 mm Hg; *p* < 0.001) and diastolic blood pressure—DBP (−4 mmHg; *p* < 0.001) among 58 poorly controlled patients with T2D, compared with the placebo [107]. A randomized, double-blinded clinical study of 66 Chinese with T2D treated with gliclazide found significant reductions in HbA1c in those receiving 360 mg of cassia cinnamon extract (−0.92%; *p* = 0.0004) and 120 mg of cassia cinnamon extract (−0.67%; *p* = 0.003) after 90 days. No significant reductions were seen in the placebo group but the groups were not compared statistically. Fasting glucose levels were also significantly reduced over time in both treatment groups [106]. A study [116] in 60 subjects with T2D treated with sulfonylureas found significant reductions in fasting glucose (18%–29%), triglycerides (23%–30%), LDL (7%–27%), and total cholesterol (12%–26%), following supplementation with either 1, 3, or 6 g of cassia cinnamon daily for 40 days, while no change was observed in the placebo group but the groups again were not compared statistically. Significant effects were observed even after 20 days of washout [116]. A double-blind study [117] found that intake of 3 g daily cinnamon powder for 120 days significantly decreased fasting glucose but not HbA1c in 65 patients with T2D treated with oral antidiabetics or diet alone. Fasting glucose was reduced by 10.3% ± 13.2% in the treatment group and 3.37% ± 14.2% in the placebo group (*p* = 0.046). No significant changes in HbA1c, total cholesterol, low-density lipoprotein (LDL), high-density lipoprotein (HDL) or triglycerides (TG) were observed. Baseline HbA1c values were below 7% [117]. Two recently updated meta-analyses showed that the intake of cinnamon/cinnamon extracts lowered fasting glucose levels [123,124]. A meta-analysis of 10 randomized controlled trials (543 patients) reported that the consumption of cinnamon ranging from 120 mg/day to 6 g/day for 4–18 weeks decreased levels of fasting glucose, total cholesterol, LDL, and triglyceride and increased levels of HDL. No significant effect on HbA1c was observed [124]

In summary, the consumption of cinnamon may have glycaemic-lowering effects, but more intervention studies covering different types and amounts of cinnamon and types of subjects, are required as not all studies have shown positive effects of cinnamon on insulin resistance and many have been poorly analysed. The usual dose of cinnamon used was 1–2 g/day but some studies used larger doses of 6 g/day which would be difficult to maintain long term.

### 5.4. Grape Polyphenols

A double-blinded, randomized, crossover trial of 32 subjects with T2D showed positive effects of grape seed extract intake of 600 mg/day for four weeks on fructosamine, whole blood glutathione (GSH; *p* < 0.01), high sensitivity C reactive protein hsCRP, (*p* = 0.0006) and total cholesterol concentration (*p* = 0.05), but did not show significant changes in HOMA–IR [125]. Another study showed protective effects of grape polyphenols on fructose-induced oxidative stress and insulin resistance in first-degree relatives of patients with T2D. Subjects were randomised to eight weeks of supplementation with 2 g/day grape polyphenols or placebo and then ingested 3 g fructose/kg fat free mass/day as a 20% fructose solution with the three meals during the six days preceding muscle biopsies and a two-step hyperinsulinemia-euglycemic clamp. After six-day fructose loading, the placebo group showed a 20% decrease in hepatic insulin sensitivity (11.9 ± 1.5 *vs.* 9.6 ± 0.9; *p* < 0.05) associated with an 11% decrease in glucose infusion rate (*p* < 0.05). However, the grape polyphenol group did not show these deleterious effects of fructose. Moreover, grape polyphenol supplementation showed protective effects against fructose-induced oxidative stress markers of urinary F2-isoprostanes, muscle thiobarbituric acid reactive substances (TBARS), while fructose load induced a downregulation of mitochondrial genes and decreased mitochondrial respiration in permeabilized muscle fibers [126]. In a randomized, controlled study of 38 males with at least one component of metabolic syndrome [127], the daily consumption of 20 g of wine grape pomace flour containing 10 g of dietary fiber, 822 mg of polyphenols for 16 weeks showed a significant reduction (*p* < 0.05) in postprandial insulin and fasting glucose levels compared with the baseline, but there were no significant differences in fasting insulin, postprandial glucose, insulin, glycosylated haemoglobin and HOMA–IR between a wine grape pomace flour group and a control group [127].

In summary, there is little persuasive evidence of an effect of mixed grape polyphenols on glucose homeostasis.

Resveratrol (3, 5, 4′-trihydroxy-trans-stilbene) is a phytoallexin naturally synthesized by plants in response to infection and injury pathogens such as bacteria or fungi. Berries, grape skins, red wine, Japanese knotweed, peanuts, and the roots of rhubarb are sources of resveratrol. Resveratrol exists in two isomeric forms. The main form is trans-resveratrol mainly found in red grape juice (3.38 mg/L) [128]. The levels of resveratrol in food vary. Its levels in red wine are higher (5.8 mg/L *vs.* 0.2 mg/L) than white wine as red wine is fermented with skins [129].

A randomized, double-blind, crossover study of 11 healthy, obese men showed that resveratrol supplementation of 150 mg/day for 30 days reduced glucose, insulin, HOMA–IR and leptin, decreased inflammatory markers (TNF-α, leukocyte count, alanine aminotransferase) and SBP by 5 mmHg, improved muscle mitochondrial respiration on a fatty acid-derived substrate from the vastus lateralis muscle biopsy (~30 mg), and decreased adipose tissue lipolysis and plasma fatty acid and glycerol in the postprandial state [130]. However, there was no control group or placebo period. Nineteen subjects with T2D took 5 mg trans-resveratrol daily for four weeks and showed improved insulin sensitivity as assessed by HOMA–IR, decreased glucose levels and a delayed glucose peak following a standard meal. There was no control group or placebo used. This study considered these favourable effects were attributed to decreased oxidative stress leading to more efficient insulin signalling via the Akt pathway, with an increase in the ratio of phosphorylated *vs.* total Akt (pAkt: Akt) ratio in platelets [131]. However, a combination of other polyphenolic compounds found in red wine may contribute to the many beneficial effects, as resveratrol is known to have low bioavailability [104].

In summary, it is hard to conclude that resveratrol has anti-diabetic effects. More clinical trials of resveratrol are required to confirm the findings described above in this article.

### 5.5. Red Wine

In a randomized, controlled study, red wine significantly improved insulin sensitivity, lipid profiles and endothelial function compared with other alcoholic beverages [132,133].

A study [134] comparing the acute effects of red wine, beer and vodka on oxidative stress reported that only red wine demonstrated protection against oxygen-induced oxidative stress, while all three alcoholic beverages provided similar protection against oxygen-induced (via hyperoxia) increase in arterial stiffness. Ten males randomly consumed one of four tested beverage of red wine, vodka, beer (0.32 g ethanol/kg body weight) and water (control). Every beverage was consumed once, a week apart in a cross-over design. The volunteers breathed 100% normobaric O2 between 60th and 90th min of 3-h study protocol. Plasma lipid peroxides (LOOH—biochemical marker of oxidative stress) and uric acid (UA) concentration, blood alcohol concentration (BAC) and arterial stiffness—indicated by augmentation index (AIx)—were measured before and 30, 60, 90, 120 and 180 min after beverage consumption [134]. Moreover, 2 g/day of grape seed extract (1 g of polyphenols) also improved endothelial function in subjects with high vascular risk [135].

However, overall, there is little evidence of an effect of wine polyphenols on glucose and insulin.

### 5.6. Pomegranate

Fresh pomegranate juice is comprised of phenolic acids, including gallic acid, chlorogenic acid, caffeic acid, ferulic acid, and coumaric acids, as well as flavonoids, such as catechin, phloridzin, and quercetin. Other flavan-3-ols such as epicatechin and epigallocatechin are present in pomegranate [136]. Various *in vivo* and *in vitro* studies have demonstrated favourable effects of pomegranate on insulin sensitivity, inhibition of a-glucosidase and reduced oxidative stress and lipid peroxidation [136,137]. Very limited human studies have focused on the effects of pomegranate juice on T2D [137]. One hundred and twenty milliliters of pomegranate juice/day in obese subjects did not modify insulin secretion and sensitivity after one month of administration in a fasted state [138]. Similarly, 50 mL of pomegranate juice/day for four weeks in subjects with T2D induced no changes in fasting glucose, insulin and HbA1c [139]. Overall, there appears to be little benefit of pomegranate juice.

### 5.7. Berries

In a randomized, cross-over, double-blind, placebo-controlled acute meal study of 20 healthy subjects, no significant effects were observed on plasma glucose, plasma triacylglycerol, plasma non esterified fatty acids, as well as markers of vascular inflammation, intercellular adhesion molecule-1 (sICAM-1) and vascular cell adhesion molecule-1 (sVCAM-1) after consumption of a black currant (20% juice), compared with consumption of placebo juice devoid of polyphenols and Vitamin C. Both drinks contained 50% citric acid solution, 1% aspartame and 8% acesulfame K. However, an increase in plasma insulin levels was detected [140]. Freeze-dried blueberry beverage containing 50 g of blueberry powder (equivalent to approximately 350 g fresh blueberries) was given daily to 48 individuals with metabolic syndrome for eight weeks in a randomized controlled trial. The serum glucose concentration, lipid profiles, fasting plasma hs-CRP, IL-6, adiponectin, sICAM-1 and sVCAM-1 were not changed significantly, whereas blood pressure decreased in the blueberry-supplemented group compared with the 960 mL water control group [141]. Insulin sensitivity improved more in a 22.5 g blueberry group (22.2% ± 5.8%) than in the placebo group (4.9% ± 4.5%) (*p* = 0.02) as measured by a high-dose hyperinsulinemic-euglycemic clamp [142]. The thirty-two obese, nondiabetic, and insulin-resistant participants of this study showed no significant changes in adiposity, energy intake, and inflammatory biomarkers.

Polyphenol-rich berries may reduce sucrose digestion and absorption leading to delayed glycaemic response [143,144]. In a randomised, controlled, cross-over study of two 3-h meal tests in healthy subjects, a berry puree (150 g) made of equal amounts (37.5 g) of bilberries, blackcurrants, cranberries and strawberries, and sweetened with 35 g sucrose lowered glucose levels at 15 (*p* < 0.05) and 30 min (*p* < 0.01) compared with the control meal including 250 mL water, 35 g sucrose, 4.5 g glucose and 5.1 g fructose which are similar in glycemic profile and amounts of available carbohydrates. The berry meal and the control meal showed the peak glucose concentration at 45 and 30 min, respectively. No significant difference in the 3-h area under the glucose response curve was observed. These findings indicated that berries rich in polyphenols delayed digestion of sucrose and/or absorption of glucose from the berry meal or slowed gastric empting [144]. In three randomised, controlled, cross-over, 2-h meal studies [143], healthy female volunteers underwent four 2-h meal tests on separate visits, at least three days apart with a mix of berries and bread strawberries, and a berry mix reduced insulin but not glucose to both wheat and rye breads [143].

A randomized, single-blind, placebo-controlled, cross-over trial in an acute-meal setting showed beneficial effects of polyphenolic- and antioxidant-rich strawberry (Fragaria) on postprandial inflammation and insulin sensitivity. Twenty-four overweight subjects consumed two isocaloric meals of high-carbohydrate, moderate-fat (HCF) accompanied by either a strawberry or a strawberry-flavoured beverage that served as a placebo. The strawberry beverage significantly attenuated the postprandial inflammatory response as measured by hsCRP and IL-6 (*p* < 0.05) and reduced postprandial insulin response (*p* < 0.05). In addition to polyphenolic compounds, Vitamin C and glutathione were also suggested to contribute to the antioxidant activity of strawberry. The test meals including strawberry and placebo beverage did not contain any soluble fibre [145]. The same team conducted an extended study [146] to investigate the effects of six weeks of strawberry consumption (~95 mg total strawberry phenols/day) on HCF meal-induced increases in glucose, insulin and indicators of inflammation and homeostasis over 6 h. Twenty-four overweight subjects consumed either a strawberry beverage or a placebo beverage for six weeks after a seven-day run-in of low/no berry consumption. Strawberry consumption attenuated postprandial plasminogen activator inhibitor-1,plasminogen activator inhibitor-1 (PAI-1) (*p* = 0.002), IL-1 β (*p* = 0.05) with moderate suppression of IL-6, but no significant changes in platelet aggregation, hsCRP, TNF-α, insulin, or glucose were observed compared with the placebo [146]. Consumption of a beverage of 50 g freeze-dried strawberries (2006 mg polyphenols) for eight weeks decreased total cholesterol and LDL-C, small LDL particles and VCAM-1, whereas waist circumference, SBP/DBP, fasting glucose, triglycerides, and HDL-C were not affected, compared with control (equivalent amounts of water) in 27 obese subjects with metabolic syndrome after eight weeks [147]. Daily consumption of two cups of a strawberry drink for four weeks in women with metabolic syndrome (each cup had 25 g of freeze-dried strawberry powder equivalent to approximately 3.5 cups or 500 g fresh strawberries) decreased total cholesterol and LDL-C levels by 5% and 6%, respectively (*p* < 0.05) and decreased lipid peroxidation in the form of malondialdehyde and hydroxynonenal by 14% (*p* < 0.01) at four weeks *versus* baseline. There was no control group or control period. This study suggested that phytosterol, dietary fiber (8 g/day), or polyphenolic flavonoids content of strawberries might contribute to the hypocholesterolemic effects of freeze dried strawberry powder [148].

A small study suggested that dried cranberries might lower the glycemic response for people with T2D [149], but other studies did not find glucose-lowering effects, despite antioxidant, anti-inflammatory, and HDL-raising and LDL-lowering effects of cranberry juice supplementation [150,151,152,153].

In summary, berries appear to reduce the insulin response to a glucose load and have anti-inflammatory effects. However, the results are very mixed and there is limited data.

### 5.8. Extra Virgin Olive Oil

In the PREDIMED study of 3541 patients with high cardiovascular risk with a 4.1-year follow-up period, a Mediterranean diet rich in extra virgin oil showed a 40% reduction (95% CI 15–57) in the risk of T2D compared with the control group [154]. An olive-rich Mediterranean diet also improved glucose metabolism and reduced body weight in subjects with T2D. Insulin resistance was measured by HOMA–IR, adiponectin/leptin and adiponectin/HOMA–R ratios after one year of follow-up, but HbA1c was not measured [155]. Moreover, the Mediterranean diet supplemented with virgin olive oil or nuts showed an anti-inflammatory effect by decreasing CRP, IL-6 and adhesion molecules, chemokines and T-lymphocytes (CD49d) and monocytes (CD 11b, CD49d, CD40) [156]. In contrast, in a study of a six-week olive oil supplementation in healthy adults, fasting glucose and HDL-C increased compared with baseline in both the low- and high-phenolic olive oil group. There was no change in fructosamine, or plasma total phenols within either group. No significance in a proteomic biomarker score for diabetes was observed with either low- or high-phenolic olive oil [157]. Phenolic components such as oleuropein and hydroxytyrosol abundant in olive leaves as well as oil may protect against diabetes and metabolic disorders [158]. Only two intervention studies of olive leaf polyphenols [159,160] have been conducted so far. Supplementation with olive leaf polyphenols (51.1 mg oleuropein and 9.7 mg hydroxytyrosol per day) for 12 weeks significantly improved insulin sensitivity and pancreatic β-cells secretory capacity after oral glucose challenge in overweight, middle-aged men at the risk of developing the metabolic syndrome [159]. Similarly, in a randomized, placebo-controlled trial in subjects with T2D, supplementation of a 500 mg olive leaf extract tablet once daily for 14 weeks was shown to significantly lower HbA1c and fasting insulin with no significant changes in postprandial insulin levels [159,160].

More clinical studies evaluating the potential effects of olive oil or leaf polyphenols on insulin sensitivity and glycaemic control should be conducted to confirm these beneficial effects.

### 5.9. Whole Grains, Nuts and Seeds

Whole grains such as wheat, rye, soy and flaxseed are a good source of polyphenols as are nuts such as chestnuts, hazelnuts, pecans and almonds [161]. Whole grain intake is associated with a reduced risk of T2D but the mechanism of the protection is not clear [162]. Whole grain intake is also associated with a lower body weight, lower waist circumference and lower fasting insulin in the Framingham offspring study [163]. However, interventions comparing whole grain with refined grain cereals for 12 weeks have shown no effect on insulin sensitivity or insulin release in 146 individuals with the metabolic syndrome [164] although 2- and 3-h postprandial insulin and triglyceride was lower after the whole grain intervention [165] compared with a standard test meal during the run-in period. A large study with 316 individuals compared usual diet (<30 g/day of whole grains) to 60 and 120 g/day for four months. No effects were seen on glucose, insulin, inflammatory, coagulation or endothelial markers [166]. It would appear despite the epidemiology that increasing whole grain intake with more fibre and polyphenols has little effect on metabolic risk. However, in the Predimed study, nuts reduced the incidence of T2D assessed annually by OGTT by 52% in the Reus Substudy over four years [167].

### 5.10. Fruits and Vegetables

Most prospective studies have shown no effect of vegetables and fruits on insulin resistance or the risk of T2D [168,169,170,171]. Only a high daily consumption of green leafy vegetables was inversely associated with T2D [169,170], while a high intake of berries was associated with a 35% lower risk of T2D (HR 0.65; 95% CI 0.49–0.88; *p* = 0.15) [171].

### 5.11. Dietary Patterns

#### 5.11.1. Polyphenol-Rich Diets

Polyphenol-rich diets have been shown to reduce blood glucose in a 3-h OGTT by increasing early insulin secretion and improving insulin sensitivity. Eighty-six participants aged 35–70 years with a BMI of 27–35 kg/m^2^ at high cardiometabolic risk were randomized to one of four eight-week isoenergetic diets with a 2 × 2 factorial design [90]: (1) high in polyphenols and high in long chain n-3 polyunsaturated fatty acids (2861 mg of polyphenols/day); (2) high in polyphenols and low in long chain n-3 polyunsaturated fatty acids (2903 mg/day); (3) low in polyphenols and high in long chain n-3 polyunsaturated fatty acids from fish (363 mg/day); (4) low in polyphenols and low in long chain n-3 polyunsaturated fatty acids (365 mg/day). Polyphenols came from decaffeinated green tea and coffee, dark chocolate, blueberry jam, extra-virgin olive oil and polyphenol rich vegetables such as steamed artichokes, onions and spinach, and raw rocket. The polyphenols were 57% flavonoids (41% flavanols) and 43% phenolic acids. The high polyphenol diet decreased plasma glucose area under the curve (AUC; *p* = 0.038) and increased the early phase of insulin secretion (AUC 0–30 min; *p* = 0.048) [172].

#### 5.11.2. Nordic Diet

Scandinavian Nordic diets are considered healthy diets as they are rich in high-fibre plant foods (such as vegetables, fruits and berries). They also include whole grains, fish, rapeseed oils, nuts and low-fat dairy but are low in salt, added sugars and saturated fats [173,174,175]. This diet appears to be related to a lower risk of T2D.

Cross-sectional studies [176] and randomized clinical trials [175,177,178] of this Nordic diet showed weight loss compared with controls. In a randomized controlled trial of 88 mildly hypercholesterolaemic subjects [175], subjects who consumed a Nordic diet for six weeks had decreased insulin (−9%; *p* = 0.01) and SBP by −6.6 ± 13.2 mmHg (−5%; *p* < 0.05) compared with the control group following their usual Western diet. However, the six-week Nordic diet caused a 4% weight loss compared with the control group (*p* < 0.001). After adjustment for weight change, no significant differences in DBP or triglyceride or glucose was observed although blood lipids were still improved [175]. Similarly, a weight-stable Nordic diet for 18–24 weeks [177] did not alter insulin sensitivity and blood pressure in people with metabolic syndrome. Significant changes between the groups were found in non-HDL-C, LDL-C to HDL-C and apolipoprotein B to apolipoprotein A1 ratios favouring the Nordic diet.

In contrast, a prospective study of 10-year follow-up showed no association between a healthy Nordic diet and the risk of T2D [179]. In a systematic review [173] of prospective observational and randomized intervention studies investigating the association between five food groups (red and processed meat, whole grains, berries, milk products and potatoes) commonly consumed in Nordic countries and the risk of T2D, only wholegrain was inversely associated with the risk of T2D [173]. The association between a Nordic diet and a lower risk of increased hs-CRP levels was observed in a meta-analysis of three cross-sectional Finnish studies [180]. Nevertheless, high adherence to this diet was associated with a lower risk of low HDL-C levels, especially in women [180].

#### 5.11.3. Mediterranean Diet

There have been no interventions comparing a Mediterranean diet to a Western control diet for the prevention of T2D but there have been many interventions in people with T2D. A recent meta-analysis of nine interventions showed that a Mediterranean diet reduced HbA1c by 0.3% and also improved many other metabolic parameters [181].

## 6. Conclusions

*In vitro* and *in vivo* studies have shown that dietary polyphenolic compounds improved glucose homeostasis through potential multiple mechanisms of action in the intestine, liver, muscle adipocytes and pancreatic β-cells, as well as through prebiotic effects in the digestive tract. Overall, most epidemiological studies showed that dietary polyphenols were associated with a lower risk of T2D, but this association was not entirely consistent. Berries and cassia cinnamon may be promising candidates for diabetes prevention and management. There have only been limited clinical studies of dietary polyphenols in relation to glucose and insulin homeostasis and these have not been as successful as *in vitro* and *in vivo* studies. The mixed results may be related to poor statistical analyses, small sample size (many are acute studies) and no dynamic measures of glucose metabolism. Larger and better designed studies are required before any recommendations can be made.
